# A Statistical Model to Predict Protection Against Infant Respiratory Syncytial Virus Disease Through Maternal Immunization

**DOI:** 10.3390/vaccines12121351

**Published:** 2024-11-29

**Authors:** Bing Cai, Yili Chen, Yasmeen Agosti, Beate Schmoele-Thoma, Kenneth Koury, Kathrin U. Jansen, William C. Gruber, Philip R. Dormitzer, Kena A. Swanson

**Affiliations:** 1 Vaccine Research and Development, Pfizer Inc., Collegeville, PA 19426, USA; 2 Clinical Development and Operations, Pfizer Inc., Collegeville, PA 19426, USA; 3 Chief Medical Affairs Office, Pfizer Inc., Collegeville, PA 19426, USA; 4 Vaccine Research and Development, Pfizer Inc., Pearl River, NY 10965, USA

**Keywords:** RSV, viral infection, infants, airway resistance, modeling, vaccine efficacy

## Abstract

Background/Objectives: Respiratory syncytial virus (RSV) is the leading cause of severe respiratory disease in infants worldwide. Maternal immunization to protect younger infants is supported by evidence that virus-neutralizing antibodies, which are efficiently transferred across the placenta from mother to fetus, are a primary immune mediator of protection. In maternal RSV vaccine studies, estimates of correlates of protection are elusive because many factors of maternal–fetal immunobiology and disease characteristics must be considered for the estimates. Methods: We developed statistical models that aims to predict vaccine efficacy (VE) in infants following maternal immunization by including quantifiable covariates of the antibody titer distribution of the mother (pre- and post-immunization), the transplacental transfer ratio of IgG antibodies, the rate of antibody decay, and RSV disease incidence rate, all of which are season- and time-dependent and vary by infant age. Result: Our model shows that integrating the lower respiratory tract disease risk based on infant airway diameter and associated airway resistance is critical to appropriately model predicted infant VE. The VE predictions by our models, which preceded maternal RSV prefusion F vaccine efficacy trial primary readouts, closely align with the VE outcomes of these field studies. Conclusion: Our models successfully predicted VE of the RSV maternal vaccines and have potential use in modeling the clinical trial out-comes of other respiratory disease vaccines where maternal antibodies play a role in the protection of newborns.

## 1. Introduction

Respiratory syncytial virus (RSV) is a leading cause of infant hospitalization globally and a major cause of infant mortality in low- and middle-income countries (LMICs), especially during the first 6 months of life [1]. Hospitalization data point to the greatest disease burden occurring in newborns under 3 months of age [2]. Infants with RSV can develop an acute lower respiratory tract infection (LRTI), manifesting as bronchiolitis, which can be severe and life-threatening. Premature infants, defined as those born before 37 weeks of gestation, also have high rates of RSV-associated intensive care unit (ICU) admission and mortality [3,4]. A maternal immunization approach whereby pregnant women are immunized can boost the level of protective RSV-neutralizing IgG antibodies that mothers naturally transfer across the placenta to their fetuses to prevent infant RSV disease through at least the first six months of life [5]. The protective maternally derived IgG provided to the fetus transplacentally before birth overcomes the potential challenges of immunizing infants in time to prevent severe RSV disease, which peaks between one and two months of age [6].

The immunization of RSV-naïve infants with a formalin-inactivated RSV vaccine was previously associated with poor efficacy and vaccine-elicited disease enhancement [7], driven, in part, by CD4^+^ T-helper type 2 cellular immune responses [8]. Maternal immunization alleviates any potential theoretical risk of vaccine-mediated RSV disease enhancement, as adults have universal exposure to RSV throughout their life [9] and only IgG crosses the placenta to mediate infant protection. Passive immunoprophylaxis using RSV-neutralizing IgG monoclonal antibodies (mAbs) targeting the RSV fusion (F) protein, such as licensed mAbs palivizumab (Synagis^®^) and nirsevimab (Beyfortus^®^), have been effective and safe in preventing RSV disease in infants. In preclinical studies, the levels of protection mediated by palivizumab [10] and nirsevimab [11] correlate with the neutralizing titers of the animals’ sera, and decades of clinical and real-world evidence with palivizumab [12,13,14,15] and, recently, nirsevimab [16], support the idea that virus-neutralizing antibodies are the primary mechanism of protection.

In the pivotal, global phase 3 study (NCT04424316) evaluating the safety and efficacy of Pfizer’s prefusion F RSV vaccine (RSVpreF) against infant RSV disease, the primary analysis showed that vaccination with RSVpreF during the third trimester of pregnancy was safe and effective, lowering the risk of severe LRTI in infants by over 80% within 90 days of birth, and by 69% within 180 days of birth [17]. RSVpreF, marketed as Abrysvo^®^, is now approved for use in pregnant women to protect infants throughout the first 6 months in the US [18], Europe [19], and other countries. RSVpreF is a bivalent vaccine, containing two RSV fusion (F) glycoprotein antigens—one for each antigenic subgroup (A and B). Both antigens are stabilized via structure-based engineering in the prefusion conformation, which is optimal for eliciting RSV-neutralizing antibodies [20].

During the early development of RSVpreF, we sought to assess the probability of its success in protecting infants against RSV disease prior to advancing to late-stage clinical trials. We developed a statistical model that predicts maternal immunization vaccine efficacy (VE) against infant RSV disease based on vaccine-elicited increases in combined RSV A/B-neutralizing titers. Our model was first shared publicly in 2019 [21], soon after the initial press release on Novavax’s maternal RSV vaccine phase 3 trial [22], and again in 2019 [23], before trial immunogenicity and efficacy data were published for both Novavax and Pfizer maternal RSV vaccine candidates [24,25]. This study outlines the model framework used to predict VE outcomes against infant RSV disease and compares the model predictions to real-world data from these two independent maternal RSV bivalent prefusion F vaccine efficacy trials.

## 2. Methods

### 2.1. Statistical Model

**Figure 1:**
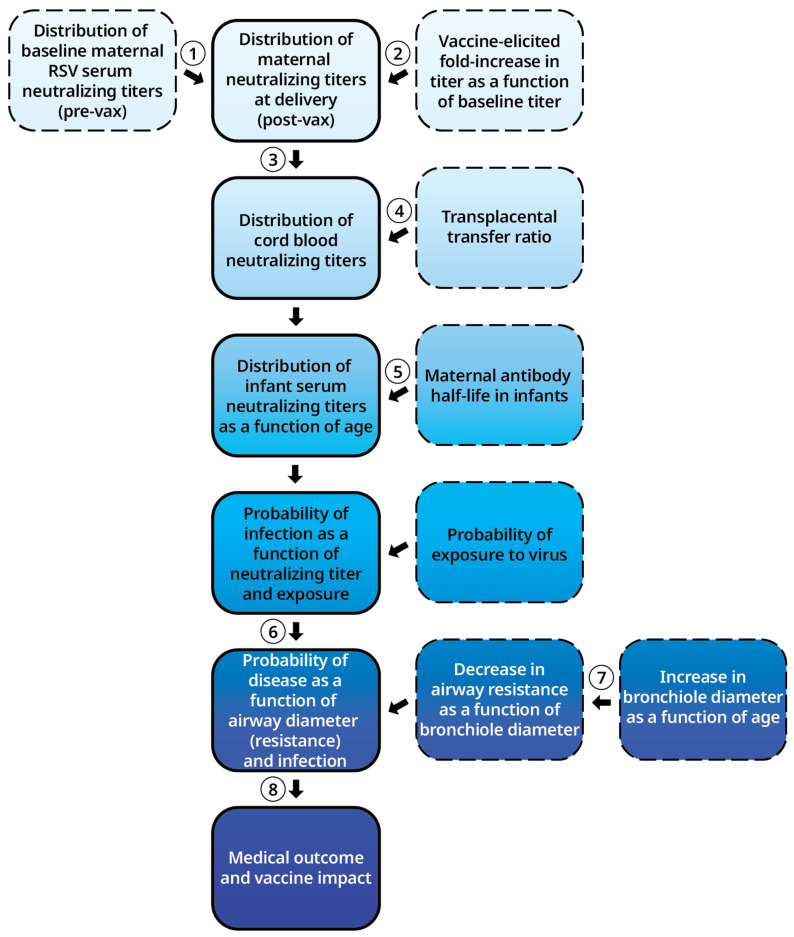
Framework of the model. This diagram depicts the multiple steps of the statistical model, the sequential order of equations used for each step, and the different assumptions or probabilities that are inputted or generated in each step. Equations (1)–(8) are circled here for emphasis.

The framework of the model (Figure 1) made the following assumptions regarding the RSV-neutralizing titers of mothers and infants, the probability of infection, and the probability of severe RSV disease. The assumptions applied to different equations in the framework, which were numbered (1)–(8):

(a) Maternal serum RSV-neutralizing titers pre- and post-vaccination.

Equation (1) assumes that the RSV-neutralizing titers on a *log* scale follow a normal distribution

*log*_2_ D_0_ ~ N (*µ*, *ơ*_1_^2^) (1)

where D_0_ is the pre-vaccination maternal neutralizing titer.

Furthermore, Equation (2) assumes a linear relationship between pre-vaccination and post-vaccination neutralizing titers on a *log* scale [26]

*log*_2_ D_1_ = *α*_2_ + *β*_2_*log*_2_ D_0_ + *ε      ε* ~ N(0, *ơ*_2_^2^) (2)

where D_1_ is the post-vaccination maternal neutralizing titer, α_2_ is the intercept, and *β*_2_ is the regression coefficient of the *log*_2_ post-vaccination neutralizing titers to *log*_2_ pre-vaccination neutralizing titers.

The model assumes that subjects with higher and lower pre-vaccination neutralizing titers will have higher post-vaccination neutralizing titers and a higher fold-rise after vaccination, respectively, based on observations made in the phase 1/2 first-in-human study of the RSVpreF vaccine [27].

(b) Transfer of maternal neutralizing antibodies to the fetus.

Equation (3) assumes that *log*_2_ maternal neutralizing titers at the time of delivery are a linear function of the *log*_2_ maternal neutralizing titers post-vaccination, and Equation (4) assumes the *log*_2_ neutralizing titers in umbilical cord serum are a linear function of the *log*_2_ maternal neutralizing titers at the time of delivery [6].

*log*_2_ D_2_ = *β*_3_*log*_2_ D_1_ + *ε    ε* ~ N(0, *ơ*_3_^2^) (3)

*log*_2_ D_3_ = *β*_4_*log*_2_ D_2_ + *ε    ε* ~ N(0, *ơ*_4_^2^) (4)

where D_2_ is the maternal neutralizing titer at delivery, D_3_ is the neutralizing titer in cord serum, *β*_3_ is the ratio of maternal neutralizing titers at delivery to the neutralizing titers post-vaccination at the log scale, *β*_4_ is the ratio that determines transplacental IgG transfer ratio at the log scale, and *ơ*_3_^2^ and *ơ*_4_^2^ are the variance in titer distribution at the *log* scale.

(c) Decay of infant neutralizing titers over time.

Equation (5) assumes that maternal neutralizing antibody titers decay exponentially over time, which means the *log* of the titers is a linear function of infant age.

$$x_{\left(t\right)}=x_{\left(0\right)}e^{\alpha_{5}t}$$

*log x*(*t*)** = *log x*(0)** − *α*5*t* (5)

where *α*_5_ is the rate of decay of maternal titers, *x*_(0)_ is the antibody titer at birth, and *x*_(*t*)_ is the neutralizing titers of infants at different ages, *t*.

(d) Probability of infection.

Equation (6) assumes that infants are exposed to RSV at a specific rate, and that if they are exposed, the probability of infection is a logistic function (*logit*) of residual, maternally derived, neutralizing titers in infant serum on a *log* scale, with a 5-day incubation period between exposure and infection.

(6) $$\mathrm{logit}\;P_{\mathrm{infection}}=\alpha_{6}+\beta_{6}\log(x_{\left(t\right)})$$

*β*_6_ is the *log* of odds ratio of infection over residual maternally derived titers in infant serum.

(e) Probability of developing severe RSV disease during RSV infection.

Equation (7) assumes that infant airway bronchiole diameter is a linear function of age. This equation was adapted from Hislop et al. [28], where infant age was measured in weeks. We converted weeks to days in our model. Equation (8) assumes that the *logit* of probability of severe disease during an RSV infection is inversely proportional to the fourth power of infant airway resistance [29].

*d* = *α*_7_ + *β*_7_(age/7) (7)

*logit* P_severity_ = ξ/(*d* − τ)^4^ + γ (8)

where age is measured in days (divided by 7 to convert weeks to days), *α*_7_ is the infant airway diameter at birth, and *β*_7_ is the increase in infant airway diameter per week. The parameters in Equation (8) were estimated by fitting the curve of the epidemiology data.

### 2.2. Model Simulations

Monte Carlo simulations were used with the parameters of each equation and withing the framework. Subjects (*n* = 10,000) were simulated with a ratio of 2 (vaccination group–placebo group). Half of the participants (*n* = 5000) were simulated using the parameters for RSV subgroup A and the other half (*n* = 5000) using the parameters for subgroup B. The results for the two subgroups were then combined to calculate incidence rate and VE.

### 2.3. Determination of Palivizumab Neutralization Titer Using Human Sera

To generate logistic dose–response curves for the protection of infants via RSV-neutralizing antibody, serum concentrations of palivizumab were translated to serum neutralizing titers by spiking multiple samples of palivizumab, with a range of concentrations, into a pooled human sera matrix and determining the RSV A- and RSV B-neutralizing titers attributable to the spiked palivizumab concentration, as described previously [30].

### 2.4. Calculation of Combined RSV A/B-Neutralizing Titers and GMFR

The combined neutralizing titers and geometric mean fold-rise (GMFR) for RSV subgroups A and B (referred to throughout as RSV A/B) were calculated based on the GMTs of RSV A and RSV B, as described previously [27].

## 3. Results

### 3.1. Model Overview (Equations (1)–(8))

The model framework consists of eight stepwise equations, summarized in Figure 1. The equation details, including specific variables and assumptions, are outlined in the Methods section. In general, the model begins by establishing a baseline population distribution of serum 50% RSV-neutralizing titers (NT_50_) in women of childbearing age (Equation (1)), and the increase in neutralizing titers post-immunization as a function of the starting baseline titer (Equation (2)). Equation (2) can be varied based on assumptions about the neutralizing antibody response to immunization. At this step, the model yields the maternal distribution of neutralizing titers at delivery (Equation (3)). An additional equation for the transplacental transfer of maternal titers provides the distribution of neutralizing titers in cord serum (Equation (4)). The model further accounts for the exponential decay of residual maternal neutralizing titers in infants (Equation (5)), yielding the distribution of infant serum neutralizing titers as a function of age.

The protective serum levels of neutralizing titers were based on the described protective serum concentrations of palivizumab [14,31,32]. Models of the probability of infection as a function of these serum neutralizing titers, combined with a model of exposure to potentially infectious doses of RSV, results in an incidence rate of RSV infection as a function of age (Equation (6)). A function for the probability of severe disease during RSV infection, based on infant airway diameter changes by infant age in months, and its effect on resistance to airflow, provides the age distribution and extent of severe RSV disease (Equation (7)). A final equation for comparing modeled outcomes with and without immunization yields insights into potential VE (Equation (8)). The outputs from each key equation are described in detail in the following sections.

### 3.2. Distribution of Baseline Maternal RSV-Neutralizing Titers

The baseline, pre-immunization distribution of maternal serum RSV-neutralizing titers is modeled as a normal distribution of *log*_2_-transformed titers (Equation (1)), matching the distribution observed in a large phase 1/2, first-in-human clinical trial of RSVpreF [27]. With the Pfizer RSV subgroup A and subgroup B neutralization assays [27,33], the baseline neutralizing titer distribution parameters are similar for the two subgroups: a mean (µ) of 10.63 and a standard deviation (ơ_1_) of 1.44 for RSV A, and a mean of 10.51 and standard deviation of 1.66 for RSV B.

### 3.3. Effect of Immunization on the Distribution of Maternal RSV-Neutralizing Titers

The parameters used to transform the pre-immunization distribution of maternal RSV-neutralizing titers to the post-immunization distribution (Equation (2)) were provided by a linear regression analysis of pre- and post-immunization titers in women of childbearing age from the first-in-human RSVpreF study [27]. That analysis provided a ratio of pre- to post-immunization, *log*_2_-transformed, RSV A-neutralizing titers (β_1_) of 0.27 and a titer distribution variance (ơ_2_) of 1.46; for RSV B, β_1_ was 0.29 and ơ_2_ was 1.19. The decay rate (α) of the increases in maternal neutralizing titers varied based on the differences in the fold-rise in maternal titers from pre- to post-immunization. This reflects the observation that a subset of vaccine recipients who had a relatively higher rate of pre-immunization neutralizing titers also developed relatively lower fold-rises, while a subset of vaccine recipients who had relatively low levels of pre-immunization neutralizing titers exhibited a higher fold-rise in post-immunization neutralizing titers (Figure S1). In the initial application of the model, the fold-rise in maternal neutralizing titers from pre- to post-immunization was varied from 2- to 28-fold (Table 1), which allowed us to model the effect that differing magnitudes of vaccine immunogenicity had on VE.

Equation (3) transforms the distribution of maternal serum RSV-neutralizing titers after immunization to the distribution of neutralizing titers at delivery and assumes a similar distribution post-immunization and at delivery. The ratio of *log*_2_-transformed third-trimester and delivery RSV-neutralizing titers (β_2_) was set to 1, with a standard deviation (σ_2_) of 0.68, based on published data on natural RSV infection in pregnant women [6].

### 3.4. Transplacental Transfer of Maternal Neutralizing Antibodies

#### 3.4.1. Transplacental Transfer Ratio of RSV-Neutralizing Titers at Delivery

Equation (4) describes the transplacental transfer of RSV-neutralizing titers from mother to fetus. The transplacental transfer ratio of cord serum titers to maternal serum titers at delivery (β3) in Equation (4) was set at 1 with a standard deviation (σ3) of 0.7 based on natural RSV infection data [6]. Based on data from an observational study of mother–infant pairs in the US and the Republic of South Africa (RSA) [30], and from Pfizer’s phase 2b study of maternal immunization with RSVpreF [25], the geometric mean transplacental transfer ratios for RSV-neutralizing titers in the US are greater than 1, and those in the RSA are less than 1. Therefore, a transplacental transfer ratio of 1 is a reasonable assumption for a global trial.

#### 3.4.2. Exponential Decay of Passively Acquired Maternal Neutralizing Antibody in Infants

Equation (5) describes the exponential decay of maternally derived serum RSV-neutralizing titers in infants from the level observed in cord serum. In this equation, the antibody decay rate (*α*), which determines the half-life of passively acquired neutralizing titers, is the key parameter. The boosting of infant serum neutralizing titers via asymptomatic RSV infections can inflate half-life estimates from the cord and infant serum neutralizing titer measurements, as was observed in the Pfizer-sponsored natural history study [30]. In the phase 2 study of the Novavax RSV vaccine in pregnant women, a half-life in infants of 34 to 36 days was observed [34]; in the context of natural infection (i.e., in the absence of vaccination), in infants in Bangladesh, a half-life of 38 days was observed [6]. Although these half-lives also could have been affected by active infant immune responses to RSV infection, they are similar to the half-lives observed for maternal antibodies against pathogens that less frequently infect neonates. For example, the reported half-life of the antibody against *Haemophilus influenzae* type b polyribosylribitol phosphate polysaccharide in infants born to unimmunized women is 35 days, and against tetanus, the antibody half-life is 32 to 42 days [35]. In unimmunized infants, the half-lives of maternal antibodies to pertussis are 36 days for lymphocytosis promoting factor, 40 days for filamentous hemagglutinin, and 55 days for pertussis agglutinins [36]. Based on this evidence, a conservative 35-day half-life, corresponding to an α of 0.0198 in Equation (5) for maternal RSV-neutralizing antibody in infants, was chosen for statistical modeling.

### 3.5. RSV Infection as a Function of Age at Different Levels of Vaccine-Elicited Increases in Maternal Neutralizing Titers

Equation (6) describes the probability that an infant is infected with RSV upon exposure to a potentially infectious dose of the virus, as a logistic function (*logit*) of residual, maternally derived neutralizing titers in infant serum on a *log* scale and with a 5-day incubation period between becoming infected and developing symptoms. An average incubation period of 5 days was assumed for this model because, although this period ranges from 2 to 8 days in infants, it is commonly 4–6 days [37]. For modeling overall global VE in a large number of infants, including those in regions where seasonal and year-round RSV circulation occur, we used a simple model employing a constant rate of exposure to virus throughout the year.

The logistic function relating neutralizing titers to probability of infection following exposure was grounded in data from the pivotal, phase 3 IMpact clinical trial, which supported the licensure of palivizumab to prevent RSV disease in high-risk infants [14]. In the IMpact study, 1502 high-risk infants, ≤6 months old, and born at ≤35 weeks gestational age, or ≤24 months old with bronchopulmonary dysplasia, were injected with monthly 15 mg/kg doses of palivizumab throughout the RSV season. The infants were then followed for the course of the study to determine the overall rate of RSV-associated hospitalization and other study outcomes among study participants. The palivizumab dosage used in the IMpact study was set to maintain trough serum levels of 40 μg/mL or greater during the at-risk period based on cotton rat infectious RSV challenge studies in which 25–30 μg/mL palivizumab suppressed virus titers in the lung by >99% [10].

An analysis of the trough titers following each dose of palivizumab, along with the relative timing of the doses and RSV season in the IMpact study, indicated that the mean trough palivizumab serum level at the peak of the RSV season was approximately 60 µg/mL [14]. Maintenance of this trough serum level resulted in a 55% reduction in RSV-associated hospitalization and a 57% reduction in pediatric intensive care unit (PICU) admissions in the high-risk group under 6 months of age. A post hoc analysis of the IMpact study showed that no infant who presented to the hospital with a palivizumab level of 92 μg/mL or greater was admitted to the PICU [31]. For modeling, we assumed a 100 μg/mL palivizumab level to be associated with protection against PICU admission.

**Figure 2:**
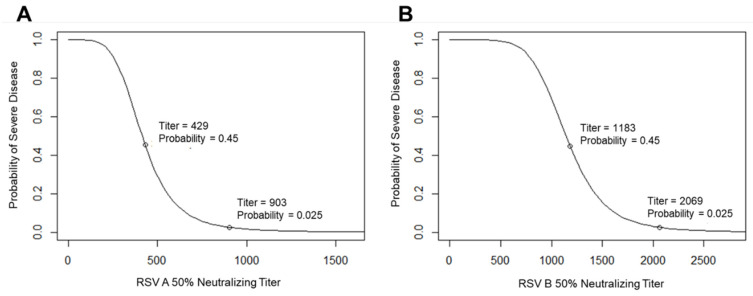
Logistic curves of the relationship between (**A**) RSV A- and (**B**) RSV B-neutralizing titers and protection against severe RSV disease. The relationship between neutralizing titers and protection against severe RSV disease is shown for (**A**) A and (**B**) B strains as logistic curves. The curves are based on observations of protection by palivizumab in the IMpact study [14], in which a prophylactic antibody was administered monthly, starting at a mean age of 5.7 months (SE 0.15) in palivizumab recipients and 6.0 months (SE 0.21) in placebo recipients [14,31]. SE, standard error.

After translating the serum concentration of palivizumab into serum neutralizing titers, logistic dose–response curves for the protection of infants using an RSV-neutralizing antibody were drawn (Figure 2) using curves anchored at the following points: (1) the RSV A-neutralizing geometric mean titer (GMT) of 903 and RSV B-neutralizing GMT of 2069, which corresponded to 100 µg/mL of palivizumab and near-complete protection (modeled as 97.5%) from RSV-associated PICU admission; (2) the RSV A-neutralizing GMT of 429 and RSV B-neutralizing GMT of 1183, which corresponded to 55% protection from RSV-associated hospitalization and 57% protection from hospitalization; (3) no protection, with a GMT of 0.

**Figure 3:**
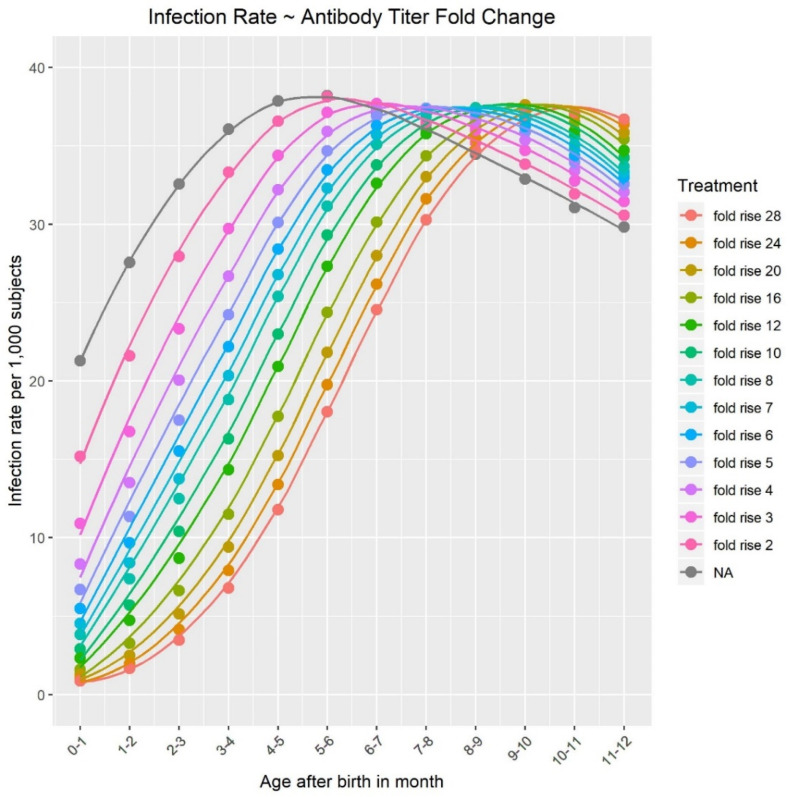
Modeled RSV infection as a function of infant chronological age at different levels of vaccine-elicited fold-rise in combined maternal RSV A/B-neutralizing titers. Each line represents the infection rate of RSV (per 1000 subjects) as a function of infant age (months) with a different vaccine-elicited GMFR of neutralizing antibody titers from the baseline. The NA (gray) line indicates no fold-rise in the absence of maternal vaccination (baseline). GMFR, geometric mean fold rise; NA, not applicable.

Ab additional modeling of these curves was needed to yield estimates of maternal VE. Using these parameters and a constant rate of exposure to virus, the model plotted the projected age distribution of RSV infection as a function of the vaccine-elicited fold-rise in maternal RSV-neutralizing titers (Figure 3). The model assumes that the incidence of RSV infection increases as the number of maternal neutralizing titers in infants falls. This phenomenon became apparent when RSV infection was modeled to peak earlier after birth in infants of unimmunized mothers compared to infants of immunized mothers, where peak RSV infection was delayed (Figure 3). For instance, a ≥20-fold rise in maternal vaccine-elicited RSV-neutralizing titers (relative to baseline) predicted RSV infection would peak at about 10 months of age—double that of infants born to unimmunized mothers. The model predicted that a higher rate of maternal neutralizing titers drastically decreases rates of infant RSV infection before 6 months of age, after which point, the pool of susceptible infants declines because a sufficient number have already been infected (Figure 3). After the first RSV infection, the model assumes that the infant will acquire active immunity; as such, the infant is subsequently removed from the susceptible pool, used for modeling purposes, after the first infection. Thus, after 6 months of age, the rates of RSV infection appeared more similar regardless of the fold-rise in maternal RSV-neutralizing antibody titers.

### 3.6. RSV Disease as a Function of Age at Different Levels of Vaccine-Elicited Increases in Maternal RSV-Neutralizing Titers

RSV infection has widely varying clinical consequences, ranging from no detectable disease to life-threatening respiratory compromise. In infants, the dominant RSV-associated clinical syndrome is bronchiolitis, an inflammation at the level of the bronchioles, which are the primary source of airway resistance [38]. The resistance of a tube to flow is inversely proportional to the fourth power of the tube’s diameter [29]. Therefore, the degree of respiratory compromise caused by bronchiolar constriction due to RSV infection is substantially dependent on the size of the bronchioles in the first few months of life. When tiny, neonatal bronchioles constrict, airway resistance can rise to fatal levels, as the resistance is too high for neonatal respiratory muscles to drive sufficient airflow to maintain oxygen intake. In-life and autopsy studies show a linear rate of growth of bronchioles from before birth through early infancy, as captured in Equation (7), with infant bronchiolar airway diameter parameters (α) of 100.4 μm at birth and a growth in airway diameter per week (β) of 1.52 μm [28]. The result of the increase in linear airway diameter during development, as captured in Equation (8), is an exponential decrease in airway resistance as an infant grows during the first months of life, and a decrease in the probability that RSV infection will cause severe respiratory compromise.

### 3.7. Validation of the Model by Comparison of Output to Real-World Observations

**Figure 4:**
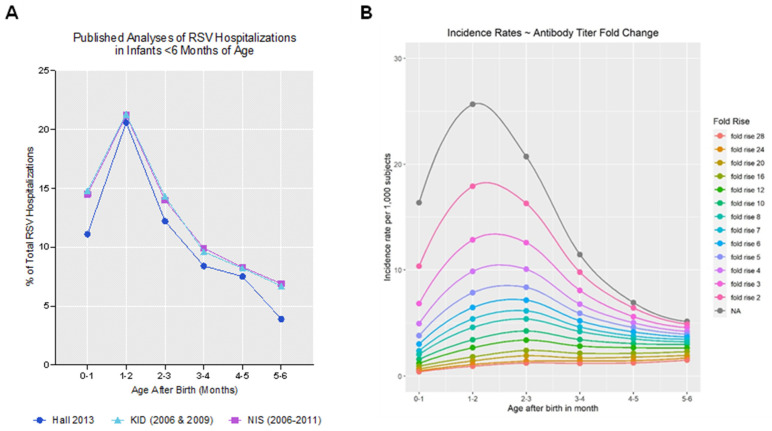
Comparison of the observed and modeled incidences of infant RSV-associated hospitalizations by chronological infant age. (**A**) Each line represents the proportion of RSV-associated infant hospitalizations by age in months from the published source in the meta-analysis by Parikh et al. [46]. Only sources in which raw data were available for each month of age (up until 6 months) were graphed. These sources included data reported by Hall et al. [45], the Kid Inpatient Database (KID) from 2006 and 2009, and the National Inpatient Sample (NIS) from 2006 through 2011. (**B**) The incidence of serious RSV disease (rate per 1000 subjects) is modeled as a function of infant age (in months) with different vaccine-elicited GMFRs of neutralizing antibody titers. Each line represents a different GMFR in the combined RSV A/B-neutralizing antibody titer elicited by a maternal RSV vaccine. The NA (gray) line indicates no fold-rise in the absence of maternal vaccination (baseline). GMFR, geometric mean fold rise; NA, not applicable.

When the age-dependence of moderate-to-severe RSV illness during the first 6 months of life was modeled based on the equations, the resulting age distribution of serious RSV disease closely matched the real-world age distribution of RSV-associated infant hospitalization (Figure 4), reported previously [39,40,41,42,43,44,45] and analyzed by Parikh et al. [46] Sources in which raw data were available for each month of age [46] were selected for comparison with our model (Figure 4A). Varying the fold-rise in maternal neutralizing titers elicited by immunization (adjusting β1 in Equation (2)) allowed for the impact of vaccine immunogenicity on the level and duration of VE against serious RSV disease in infants born to immunized mothers to be modeled (Figure 4B). Table 1 provides the modeled maternal VE levels and durations in infants as a function of vaccine immunogenicity. These projections were expressed as the elicited, combined RSV A/B geometric mean fold-rises (GMFR) between maternal pre- and post-immunization titers (one month relative to baseline) or, equivalently, as the RSV A/B geometric mean ratios (GMR) of the cord serum titers of infants born to vaccinated women (relative to women who received placebo).

The application of the model to immunogenicity data from the Novavax maternal immunization phase 3 trial (NCT02624947) validated its ability to predict VE outcomes. The vaccine candidate was lacking primary efficacy endpoints but provided some reduction in RSV-associated moderate-to-severe infant LRTI and hospitalizations [24]. The combined maternal RSV A/B-neutralizing titer GMFR, elicited by ResVax in the phase 3 trial, was calculated to be 2.2 at delivery (RSV A, 2.28; RSV B, 2.07). The cumulative efficacy over 0 to 90 days of life against moderate-to-severe RSV LRTI was 39.4% (95% CI: 5.3, 61.2) and the efficacy against RSV hospitalizations was 44.4% (95% CI: 19.6, 61.5) (Table 2). The model predicted that a vaccine that elicits a maternal RSV A/B-neutralizing titer GMFR between 2 and 3 will have a cumulative efficacy in infants from 0 to 3 months of age of 24–43% (Table 1). Thus, using only trial immunogenicity data, the model provided a conservative VE output that was similar to the real-life outcome.

In contrast to the Novavax phase 3 trial, Pfizer’s phase 2b study of maternal immunization with RSVpreF (NCT04032093) was not powered to measure an efficacy outcome; however, efficacy against infant RSV disease was an exploratory endpoint [25]. The maternal neutralizing RSV A/B GMR at delivery (relative to placebo) for the 120 μg dose of RSVpreF without Al(OH)_3_ was 12.4. Based on eight infant cases, VE corresponded to observed efficacies of 84.7% (95% CI, 21.6–97.6%) against medically attended RSV LRTI, and 91.5% (95% CI, −5.6–99.8%) against severe RSV LRTI, over a reporting period of approximately 6 months after birth (Table 2). Based on a maternal neutralizing GMFR of 12 and 16, the model generated a conservative VE cumulative over 6 months of approximately 77% and 83%, respectively (Table 1), which was close to the mean real-life VE outcomes observed in the phase 2b study.

## 4. Discussion

The well-defined mechanism of action of protection against RSV disease via a passively transferred neutralizing antibody served as our foundation for modeling infant age-specific RSV disease risk and VE following maternal immunization. The durable effectiveness of palivizumab at concentrations of 60 µg/mL or more, as demonstrated in the pivotal IMpact-RSV Study Group trial [14], together with the palivizumab logistic curves, provided the initial key inputs to establish the relationship between effective antibody concentrations, virus-neutralizing titers, and protection against severe RSV disease in infants (Figure 2). The IMpact trial analyzed a consistent, weight-adjusted dose (15 mg/kg) of palivizumab, an RSV-neutralizing antibody that was administered monthly to high-risk infants, starting at approximately 6 months of age [14]. In the study, the levels of infant neutralizing GMTs were generally low before the first palivizumab injection because few maternal antibodies persisted to 6 months of age, particularly in a population containing a high proportion of premature infants [14]. In addition, few infants were previously infected with RSV, which would generate active immunity, mainly because recent RSV infection was an exclusion criterion for study participants [14]. Therefore, the dose of neutralizing mAb administered to infants was consistent regardless of the infant’s pre-dosing serology, leading to relatively homogenous antibody kinetics.

By contrast, in a maternal vaccine trial, infant antibody levels can be heterogeneous among both placebo and vaccine groups. First, women can have widely varied pre-immunization RSV titers based on their individual RSV infection histories (before or during pregnancy) [47,48]. Second, maternal vaccine trial outcomes are measured in infants for the first 6 months after birth [17,25]; therefore, the variation in maternal antibody titers that are transferred to the infant persists throughout the time at which outcomes are measured. Moreover, there are additional covariates beyond antibody concentration and neutralizing capability that could influence how the infant responds to vaccination of the mother; these factors should incorporate immunological heterogeneity in the population and the physiology of the airway in young infants as a direct function of age. Thus, these were important factors to implement in our statistical model. Our modeling adjusted for these key differences using clinically effective concentrations of palivizumab to guide the translation between RSV A/B-neutralizing titers and expected VE in maternal immunization studies. The combined RSV A/B statistics are particularly applicable, given the equivalent incidence and severity of RSV disease in each subgroup [49]. Comparing GMFRs or GMRs—measured approximately one month after a single vaccine dose is administered in the late second or third trimester—between maternal immunization studies allows for cross-study comparisons. The comparison of fold-rises (GMFR or GMR) in neutralizing titers, rather than absolute neutralizing titers, also minimizes the potential effects of differences in the neutralization assays used.

The publications of two large-scale, multicenter, global, RSV maternal vaccine trials—Novavax’s ResVax and Pfizer’s RSVpreF—provided an opportunity to assess the model’s proximity to real-world maternal vaccine immunogenicity data. Our model’s predictions were consistent with the VE results of the exploratory phase 2b study of RSVpreF, a protein subunit vaccine containing the prefusion-stabilized F antigen [25], and the large, pivotal phase 3 efficacy study of ResVax, a nanoparticle vaccine containing a recombinant F antigen that is not stabilized in the prefusion conformation [24]. Regardless of the F antigen structure, our model accurately predicted the VE outcomes reported for both vaccine candidates. The outputs of our model, aligned with the VE data from these randomized control trials, established the necessary confidence in the achievement of a positive outcome to launch a global, phase 3, pivotal efficacy study for maternal immunization with RSVpreF. Indeed, the results of the RSVpreF phase 3 trial, reported in 2023 [17], supported FDA approval of the vaccine as the first maternal vaccine for the prevention of RSV disease in infants from birth through 6 months of age [18].

Our model predicts that RSV infection rates in infants of unimmunized mothers peak at 4–5 months of age, which is substantially higher and later than the peak in the rate of RSV disease, which is projected to peak at 1–2 months of age in those same infants. The actual RSV-associated hospitalization rates in young infants reported in the US [46] support our model’s proposed age distribution of RSV disease. In a natural history study in pregnant women and their infants in the US and South African by Madhi et al. [30], asymptomatic RSV infection was common in early infancy, with a potentially much higher overall RSV exposure rate and a higher degree of asymptomatic RSV infection in RSA versus US infants during the RSV surveillance period. Asymptomatic infections could elicit active infant immunity in the presence of maternally transferred RSV-neutralizing titers, which may offer added protection against future symptomatic RSV infections. Maternal RSV vaccine-elicited increases in neutralizing antibody titers were shown to improve the outcome for infants by delaying or reducing the incidence of RSV infection and disease, respectively. The delay in peak RSV infection beyond 6 months of age is clinically important, as an infant’s immune system has matured by this time, their airways are bigger, and thus the infant can better cope with infections. Our model highlights how maternal immunization can help lower the risk of infection in newborns, who are more vulnerable to severe RSV disease.

Although the model supported predictions that aligned closely with the observed infant VE, the model did not include all potential variables that may influence protection against infant RSV disease (e.g., the timing of maternal vaccination relative to infant birth). Further refinements that may continue to improve the model include the addition of predictions related to the impact of seasonal effect on incidence rate of severe RSV disease and VE. Modeling an exposure rate with seasonal patterns could yield predictions that could be used to support the recruiting strategy of maternal vaccine clinical trials. Elaborations of this model were specifically used for the following: (1) to tailor the timing of the maternal immunization of RSVpreF at sites with different RSV epidemiology; (2) to set the number of participants in the RSVpreF phase 3 trial; and (3) to respond to the shifting RSV epidemiology brought about by social distancing during the COVID-19 pandemic, which unfolded contemporaneously with the phase 3 trial of RSVpreF. The model assumes a similar distribution of maternal RSV-neutralizing titers post-immunization and at delivery, which may not account for the potential variability in real-world timings from vaccination to delivery. The model could also examine the effects of RSV vaccination in subsequent pregnancies and the effects of preterm delivery on maternal VE by incorporating different transplacental transfer ratios together with antibody titer at delivery.. Finally, the VE model could be extended to maternal vaccines targeting different pathogens, such as group B streptococcus (GBS), for which there is an association between higher maternal/infant anti-capsular polysaccharide IgG concentrations and protection against infant GBS disease [50].

## 5. Conclusions

In summary, we show that a focus on infant airway physiology and the dynamics of the maternal RSV antibody response are critical to providing robust, mathematical predictions of maternal RSV vaccine efficacy. The model underscores the critical role of maternal antibodies in safeguarding infants against RSV-associated disease and has potential use in modeling the clinical trial outcomes of other respiratory disease vaccines where maternal antibodies play a role in the protection of newborns.

## Figures and Tables

**Table 1:** Cumulative VE by infant month of age as a function of maternal RSV A/B-neutralizing titer GMFRs, based on a model of real-world observations.

	RSV A/B-Neutralizing Titer (GMFR) ^a^												
Infant Age (Month)	2x	3x	4x	5x	6x	7x	8x	10x	12x	16x	20x	24x	28x
0–1	0.32	0.53	0.65	0.72	0.78	0.82	0.85	0.89	0.91	0.94	0.96	0.97	0.98
0–2	0.28	0.47	0.60	0.67	0.73	0.77	0.8	0.85	0.88	0.92	0.94	0.96	0.97
0–3	0.24	0.43	0.55	0.62	0.68	0.73	0.76	0.81	0.85	0.89	0.92	0.94	0.95
0–4	0.22	0.40	0.51	0.59	0.65	0.69	0.73	0.79	0.82	0.87	0.90	0.92	0.94
0–5	0.21	0.38	0.49	0.56	0.62	0.67	0.70	0.76	0.80	0.85	0.89	0.91	0.92
0–6	0.20	0.36	0.47	0.54	0.60	0.64	0.68	0.73	0.77	0.83	0.87	0.89	0.91

Abbreviations: GMFR = geometric mean fold rise; VE = vaccine efficacy. ^a^ Cumulative VE was modeled using immunogenicity data reported from the RSVpreF first-in-human phase 1/2 study [27].

**Table 2:** Comparison of model-predicted VEs to the observed VEs reported from two RSV maternal vaccine clinical trials.

Observed					Predicted ^a^		
Trial, Sponsor, and Study	RSV A/B Vaccine Candidate	Combined Maternal RSV A/B-Neutralizing Titer at Delivery (95% CI)	Efficacy End Point	Reported VE (95% CI)	Combined Maternal RSV A/B Neutralizing Titer (GMFR)	Age of Infant	Predicted VE
NCT02624947 Novavax Phase 3	ResVax 120 µg (with aluminum adjuvant)	2.2 GMFR	0 to 90 days without moderate-to-severe RSV LRTI	39.4% (5.3%, 61.2%)	2	0–3 months	24%
			0 to 90 days without RSV hospitalization	44.4% (19.6%, 61.5%)	3		43%
NCT04032093 Pfizer Phase 2b	RSVpreF 120 μg (no adjuvant)	12.4 (9.5, 16.2) GMR	0–6 months without medically attended RSV LRTI	84.7% (21.6%, 97.6%)	12	0–6 months	77%
			0–6 months without severe RSV LRTI	95.5% (−5.6%, 99.8%)	16		83%

Abbreviations: GMFR = geometric mean fold rise; GMR = geometric mean ratio; VE = vaccine efficacy. ^a^ Model predictions were shared publicly in 2019 [21,23], prior to the publication of the Novavax and Pfizer maternal RSV vaccine clinical trials in 2020 [24] and 2022 [25], respectively.

## Data Availability

Cumulative maternal VE was modeled using data from the RSVpreF phase 1/2 first-in-human study (NCT03529773). Participant data and informed consent were previously described for this clinical trial [27]. Contact the corresponding authors for more information.

## Supplementary Materials

The following supporting information can be downloaded at: https://www.mdpi.com/article/10.3390/vaccines12121351/s1, Figure S1: RSV A/B neutralizing titer GMFR one-month post-immunization as a function of subjects’ pre-immunization RSV A/B neutralizing titers.
