# Supplementary material for: A Statistical Model to Predict Protection Against Infant Respiratory Syncytial Virus Disease Through Maternal Immunization
Source: Vaccines (Basel). 2024 Nov 29;12(12):1351. doi: 10.3390/vaccines12121351 (PMC11679883; doi:10.3390/vaccines12121351)
Supplement: Supplementary file 1 [file vaccines-12-01351-s001.zip › vaccines-3279458-supplementary.pdf]

Supplementary Materials

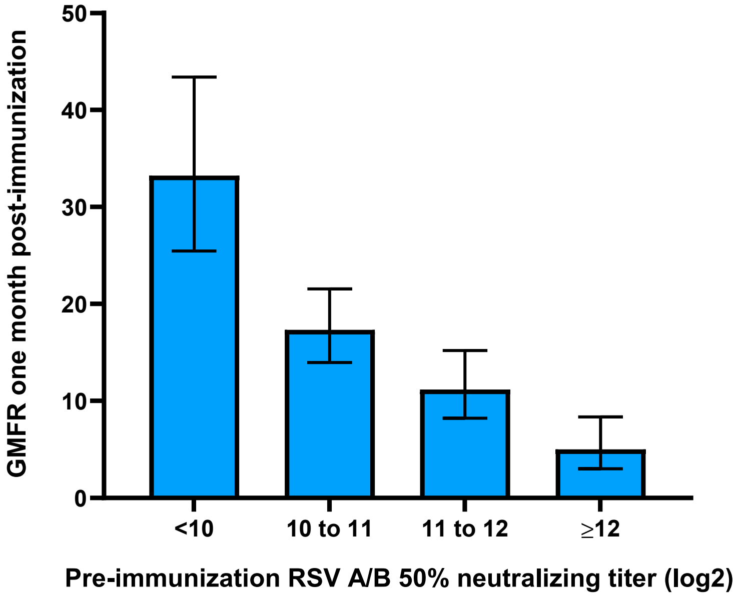

Figure S1. RSV A/B neutralizing titer GMFR one-month post-immunization as a function of subjects' pre-immunization RSV A/B neutralizing titers. The GMFR in neutralizing serum antibody titer against RSV A/B are shown one-month post-immunization with RSVpreF categorized by the subjects' respective pre-RSVpreF immunization neutralizing antibody titer on a log scale. Anti-RSV A/B neutralizing titers were obtained from adult females, aged 18-49 years old, who received 120 or 240 µg of RSVpreF without concomitant influenza vaccine in the first-in-human study of the RSVpreF vaccine.[27] GMFR, geometric mean fold rise.

Reference:

27. Walsh, E.E.; Falsey, A.R.; Scott, D.A.; Gurtman, A.; Zareba, A.M.; Jansen, K.U.; Gruber, W.C.; Dormitzer, P.R.; Swanson, K.A.; Radley, D.; et al. A Randomized Phase 1/2 Study of a Respiratory Syncytial Virus Prefusion F Vaccine. J Infect Dis 2021, doi:10.1093/infdis/jiab612.
